# Surface Modification of Lightweight Mortars by Nanopolymers to Improve Their Water-Repellency and Durability

**DOI:** 10.3390/ma13061350

**Published:** 2020-03-17

**Authors:** Małgorzata Szafraniec, Danuta Barnat-Hunek, Małgorzata Grzegorczyk-Frańczak, Maciej Trochonowicz

**Affiliations:** Faculty of Civil Engineering and Architecture, Lublin University of Technology, ul. Nadbystrzycka 40, 20-618 Lublin, Poland; d.barnat-hunek@pollub.pl (D.B.-H.); m.grzegorczyk@pollub.pl (M.G.-F.); m.trochonowicz@pollub.pl (M.T.)

**Keywords:** hydrophobisation, surface free energy, frost resistance, lightweight mortars, nanopolymers, micro-roughness

## Abstract

The paper explores the possibility of covering the mortar with the lightweight aggregate by the nanopolymer silane and siloxane as surface hydrophobisation. The investigation involved the mortars with two types of hydrophobic agents diluted with water in a ratio of 1:4 and 1:8. Mortar wetting properties were determined by measuring the absorbability, water vapor diffusion, contact angle (CA) and surface free energy (SFE) of their structure. Surface micro-roughness and 2D topography were evaluated. Scanning electron microscopy (SEM) has shown the microstructure and distribution of pores in mortars. The reduction in absorbency after the first day of testing by 87% was shown. An improvement in frost resistance after 25 cycles by 97% and an 18-fold decrease in weight loss after the sulphate crystallization test were observed. The hydrophobic coating reduces the SFE of mortars and increases the CA. In the case of using silanes, a 9-fold increase CA was observed.

## 1. Introduction

Over the last few years, researchers have been looking for more effective and ecological ways to protect building structures from a reduction in their durability. They try to modify the properties of building materials to make them more efficient and sustainable. Due to increasing environmental awareness, they are looking for solutions that will fully fit into the developing trend. Achieving long durability of building materials will contribute to the reduction of building waste production.

In line with the global trends of energy-efficient construction, conventional mortars can be replaced by lightweight alternatives. Many researchers have focused on researching lightweight eco-mortars in which traditional components have been replaced for example by, wood by-products [1,2], waste marble dust [3], expanded cork [4], cork granulate composite [5], expanded polystyrene [6], paper sludge ash [6], expanded perlite [7,8], recycled polyurethane foam [9], polyurethane wastes and non-ionic surfactants [10], polymeric wastes [11], pumice [12], vitrified microsphere [13], waste perlite power [14], Styrofoam waste [15], ground waste glass [16], aerogel [17], vermiculite and waste polystyrene [18], recycled Etna volcanic aggregates [19], Juncus maritimus fibers [20]. As shown in Reference [1], the addition of wood waste positively influenced the improvement of some properties of mortars. Use of 5% coarse sawdust, as a substitution, reduced the thermal conductivity coefficient by about 23% in comparison with the reference mortar. Studies in Reference [12] have shown that the residual compression strength after freezing cycles for pumice aggregate (PA) was higher than for Portland cement (PC) mortar. According to Ferrándiz-Mas et al. [6], for mortars with 30% of paper sludge ash (PSA) and ground expanded polystyrene (EPS), the bulk density has been reduced by 45% compared to reference mortar. In mortars with modified composition, in which aerogel was used as an additive, an improvement in their thermal properties was noticed. This is because the aerogel prevents heat transfer and improves material permeability [17].

The above-mentioned substitutes improve many properties of the mortars. However, due to porous structure, mortars are exposed to factors which exacerbate their physical and mechanical properties [21]. Some of them are undoubtedly water and capillary force, which helps water to penetrate the mortar pores. These factors cause deterioration of mortars for example, they reduce frost resistance and contribute to the penetration of water-soluble salts into mortars [22]. Moreover, high moisture content influences on lose of mortars thermal properties. Water in the pores and capillaries, which is exposed to low temperatures and changes its state of aggregation during the freezing process can contribute to the destruction of the mortar. Besides, salts that are in the water can crystallize and cause cracks, blooms and also can contribute to the destruction of the mortar structure. The humidity in salted walls increases due to absorption of moisture from the air. After several cycles of drying and impregnation, sodium sulphate destroys building materials. Some admixture or replacements for traditional aggregates make mortars less absorbent and resistant to moisture. Giosuè et al. [23] examined mortars in which sand was replaced by wool and zeolite. Their research has shown that thanks to such a combination, moisture buffering value was 100% higher than for the reference mortar. Also adsorption capacity was 65% lower, compared to a mortar in which sand was an aggregate. Amorphous carbon powder, by-product material from refining waste sewage, used as an admixture for cement composites, reduces water sorptivity and absorption by 86%, 23%, respectively [24]. But unfortunately, not all the substitutes have that kind of property. In paper [18] it can be seen that mortars that contain vermiculite and waste expanded polystyrene have a thermal conductivity equal to 0.09 W/m·K. But at the same time as the ratio of polystyrene and vermiculite to cement increases, water absorption increases. This is also due to the high porosity of such mortars, which is up to 70% concerning the model mortars. Degirmenci and Yilmaz [12] noticed that mortars with PA show much higher resistance to freezing and sulphate resistance due to high voids content. PA mortars are also resistant to high temperatures. However, the high porosity of such mortars makes their water absorption four times higher than that of PC mortars. Mortars with unconventional aggregates such as sawdust decrease the density of mortars [25]. But on the other hand, sawdust caused an increase of capillary water absorption. The properties of natural aggregates used in light mortars extracted from quarries depend on various geological sources, petrographic features of rocks [26,27]. The capillary porosity of aggregates is very important for the cohesiveness of cement-based matrices. Magma rocks have very different capillary pores in comparison to most sedimentary and metamorphic lithotypes, therefore their influence on the characteristics of concrete is expected to be different [28].

One of the most effective ways to protect building materials from water is hydrophobisation. Thanks to hydrophobisation, porous mortars can retain the properties which they have acquired through unconventional aggregates. Currently, there are two main methods of water-repellent treatment such as hydrophobic admixture method and surface treatment method [29,30,31]. Many studies have shown that hydrophobisation has a positive effect on improving material properties. Barnat-Hunek and Łagód [32] have shown that it is possible to produce pumice-modified mortars that would be protected from moisture through hydrophobisation. Frattolillo et al. [33] have examined that hydrophobized samples of mortar showed lower thermal conductivity compared to mortars that were not protected against moisture. They also showed that measurements of water vapor permeability prove that the transpiration is more effective in mortars that have been hydrophobized. In Reference [34] has been shown that the addition of 1% of the hydrophobic agent reduced capillary absorption by 84% compared to non-hydrophobic samples. Chen et al. [29] used superhydrophobic nano-silica (SNS) as an additive to cement pastes. SNS was applied in 1%, 2% and 4% by weight of cement, which contributed to a 4.2%, 13.8% and 25.1% decrease in sorptivity, respectively. In Reference [35] it was shown that thanks to hydrophobic admixture the surface of cement mortar showed superhydrophobicity. The contact angle was 157.3° and the water absorption was reduced by about 92.51%. Hydrophobisation of lightweight mortars containing waste polyurethane foam carried out in work [36] increased frost resistance up to 84% compared to the model samples. The work of Barnat-Hunek et al. [37] is another example confirming the effectiveness of hydrophobisation which has helped to protect the mortar with expanded cork from the destructive effects of frost.

Therefore, in recent years the use of various hydrophobic agents, to minimize the decomposition rate of porous materials, has been widely discussed [36,37,38]. Furthermore, the concentration and type of the active substance in the agent constitute a very crucial factor. In the literature, there can be noted examples of different additives and admixtures that can be used for hydrophobisation, such as superhydrophobic nano-silica [29], particle-siloxane [31], silica particles in rice husk ash modified using fluoroalkyl silane [30], methyl-silicon resin [36], alkyl-alkoxy-siloxane [36], SiO_2_-CH_3_ submicron-sized [38], alkyl-alkoxy-siloxane oligomer and methyl silicone resin in an organic solvent [37], cyanoacrylates [39], polydimethylsiloxane [35], ammonium polyphosphate [40], SiO_2_/ polymethyl-hydro siloxane synthesized by sol-gel [41], Nano-SiO_2_ within organic films [42].

Following the growing tendency to modify the properties of materials at the nanoscale, researchers are increasingly turning to materials of this particle size. At present, these are the smallest molecules in molecular terms used for hydrophobisation of building materials. Nanomaterials are composed of definable components having a size range of 1 to 100 nanometers in one or more dimensions. This makes the nano-impregnates exhibit much better pore penetration properties than commonly used silicone compounds. Studies in References [43,44] have shown that hydrophobic agents with the highest efficiency consist mainly of polymer and nanopolymer compounds which are produced based on silanes, siloxanes and silicones. These include ‘new-age’ nanopolymers such as nanosilanes. Silanes are a group of compounds whose common feature is the presence of a central silicon atom to which any four substituents are attached through σ bonds.

To the best of our knowledge, there are no cases in the literature of the use of nanosilanes for hydrophobisation of the lightweight mortars. Therefore, the study focuses on the evaluation of the surface modification of lightweight mortars by nanopolymers.

## 2. Materials and Methods

### 2.1. Materials

Five sets of lightweight mortar samples consisting of the same components were prepared. One specimen set was not surface-modified by hydrophobisation, it was used as a reference set. The four remaining sample sets were surface treated with two different nanosilanes in two dilution states (1:4 and 1:8)—two sets per agent. Mortar composition per 1 m^3^ is shown in Table 1. The samples were marked with the following symbols: S—non-hydrophobized mortar; A1, A2—the type of agent used.

As a binder Portland cement CEM I 42.5R (Cemex Poland, Cement plant Chełm) was used. The cement meets the requirements according to the following standards: EN 197-1:2012 [45] and PN-B-19707:2013-10 [46]. The cement used consisted of the following components (by weight): CaO (64.41%), SiO_2_ (20.23%), Al_2_O_3_ (3.62%), Fe_2_O_3_ (4.36%), MgO (1.36%), Na_2_O (0.26%), K_2_O (0.55%), Na_2_O_eq_ (0.63%). The physical properties of cement are presented in Table 2.

As fine aggregate was used quarts sand and expanded perlite. Quartz sand (fraction 0–2 mm) was delivered from Dudzik company, Lublin, Poland. It was composed of (by weight): SiO_2_ (95.3%), Al_2_O_3_ (1.9%), Fe_2_O_3_ (0.7%), CaO (0.35%). Quarts sand had a bulk density of 2700 kg∙m^−3^.

Composition of expanded perlite (fraction 0.5–2 mm) from Perlipol, Bełchatów, Poland was as follows (by weight): SiO_2_ (72%), Al_2_O_3_ (14%), Na_2_O (3%), K_2_O (4%), Fe_2_O_3_ (1%), MgO (0.5%), CaO (1%) [47]. The physical parameters of expanded perlite are shown in Table 3.

In order to reduce the amount of water used in the production process, superplasticizer was used. It is a highly liquefying agent, based on polycarboxylates with a density of 1.06 ± 0.02 g∙cm^−3^ and pH 1–5.

### 2.2. Samples

To determine the physical and mechanical properties of mortar, cuboidal samples with the dimensions of 40 × 40 × 160 mm were prepared. Samples were made in accordance with EN 196-7:2009 [48]. After the samples were made, they were stored in air-dry conditions for 24 h. Next, they were taken out of the molds and placed in water for 28 days at 20 ± 2 °C. The specimens were then dried to a constant weight and cleaned of contaminants. Surface hydrophobisation was done by applying two layers of the preparation with a brush. After hydrophobisation, the samples were subjected to a seasoning under laboratory conditions for 7 days for polycondensation reaction.

The preparations used for surface hydrophobisation are listed below:

**A1**—agent from Evonic, Essen, Germany is an aqueous emulsion based on organofunctional silanes. It is used for waterproofing mineral, absorbent substrates. The product had the following physical properties: density 0.94 g∙cm^−3^, viscosity 15 mPa∙s, pH 6–8.

**A2**—agent from Evonic, Essen, Germany, is a water-based emulsion based on silanes. It is used to give hydrophobic and oleophobic properties to porous mineral substrates. The preparation had a density of 1.018 g∙cm^−3^, viscosity 1 mPa∙s, pH 4.

They are practically free from volatile organic components (VOC). These agents can be classified as nanopolymers because their particles have a size of about 1 nm, which is several times less than that of siloxanes or silicones (>20 nm). For most applications, the manufacturer recommends a concentration of from 5% to 20% (1 part of this agent and 1.5÷10 part of water). Both preparations in this study were diluted in demineralized water in the ratio of 1:4 and 1:8 (100 mL of hydrophobic agent per 400 or 800 mL water) [49].

### 2.3. Methods

The specific density of the hardened mortar was determined by a pycnometric method according to EN 1936:2010 [50]. The samples dried to constant weight were ground in an electric mill. The test was carried out on 10 g of samples per each pycnometer. The result of the test is the average of three results.

The bulk density of hardened mortars was determined according to the following standard 1015-10 [51]. The test was carried out on samples with the dimensions of 40 × 40 × 160 mm (6 for each batch).

In accordance with the EN 1936:2006 [50], the open porosity test was performed. The result of this test is the percentage ratio of open pores to the total sample volume.

The test of water absorption was conducted on EN 13755:2008 [52]. The test was carried out on three samples of each mortar. Samples were dried to constant mass before testing. The water absorption of mortar was measured after 1, 3, 7 and 14 days. After the test, the samples were dried with a cloth and left under laboratory conditions at a temperature of 20 ± 5 °C and relative humidity of 60 ± 5% to determine the decrease in the humidity of the mortar. The test was started immediately after the end of the absorbability test. The measurements were carried out after 1, 3, 7 and 14 days. During this time the loss of mass due to evaporation of water was determined. The percentage decrease in humidity was determined as a humidity coefficient.

Following the EN1015-11:2001 standard [53], the compressive and flexural strength tests were carried out. The flexural strength was determined on samples with the dimensions of 40 × 40 × 160 mm (3 for each mortar). The compressive strength was determined on six halves of the specimens that were obtained from the flexural strength test.

The frost resistance test was carried out using the direct method according to the following standard: PN-B 12012:2007 [54]. A total of six samples for each mortar with the dimensions of 40 × 40 × 160 mm was used. Specimens were cyclically frozen in the air at −18 ± 2 °C for 4 h and then thawed in the water at +18 ± 2 °C for 2 h. In total, 25 cycles were performed. After the last freeze-thaw (F-T) cycle, the samples were dried to constant weight and weighed to determine weight loss.

The salt crystallization resistance test was conducted in accordance with the 12370:2001 standard [55]. Samples with the dimensions of 40 × 40 × 160 mm were cut into three parts, each with the dimensions of 40 × 40 × 40 mm (6 cubic samples for each mortar). After those cubic specimens were dried and placed for 2 h in a 14% Na_2_SO_4_·10H_2_O solution. The samples were then dried for 10 h in an apparatus in which the temperature rose gradually to 105 °C. The test included 15 cycles. The samples were then stored in water for 24 h, rinsed and dried to constant weight. The average of six samples was used as the result of the salt crystallization resistance test.

The Owens–Wendt method—known as the Kaelble-Owens-Wendt method—entails the determination of dispersive and polar surface free energy (SFE) components based on the Berthelot hypothesis. This hypothesis claims that interactions between molecules of two materials, present in their surface layer, are equal to the geometric mean of intermolecular interactions within each material. The following equation admits to determine SFE [56]:

(1) $$\gamma_{s}=\gamma_{s}^{p}+\gamma_{s}^{d},$$

where: $\left(\gamma_{s}\right)$ is the total SFE, $\left(\gamma_{s}^{d}\right)$ is the dispersion component of SFE and $\left(\gamma_{s}^{p}\right)$ is the polar component of SFE.

To determine the SFE, two measurement liquids (distilled water–bipolar and diiodomethane-polar), whose surface tension and components of SFE are known, are used. Distilled water is a polar liquid as its total SFE 72.8 mJ∙m^−2^ and polar component is 51 mJ∙m^−2^ [57]. The components of SFE of diiodomethane are: dispersive—48.6 mJ∙m^−2^ and polar—2.4 mJ∙m^−2^ [57]. Components of the examined surface can be appointed from Reference [58]:

(a) polar component of SFE:
  (2) $${\left(\gamma_{s}^{p}\right)}^{1/2}=\frac{\gamma_{w}\left(\cos\theta_{w}+1\right)-2\sqrt{\gamma_{s}^{d}\gamma_{w}^{d}}}{2\sqrt{\gamma_{w}^{p}}},$$
(b) dispersion component of SFE:
  (3) $${\left(\gamma_{s}^{d}\right)}^{1/2}=\frac{\gamma_{d}\left(\cos\theta_{d}+1\right)-\gamma_{w}\left(\cos\theta_{w}+1\right)\sqrt{\frac{\gamma_{d}^{p}}{\gamma_{w}^{p}}}}{2\left(\sqrt{\gamma_{d}^{d}}-\sqrt{\frac{\gamma_{d}^{p}\gamma_{w}^{d}}{\gamma_{w}^{p}}}\right)},$$
  where: $\left(\gamma_{d}\right)$ is the SFE of diiodomethane, $\left(\gamma_{d}^{d}\right)$ is the dispersive component of diiodomethane SFE, $\left(\gamma_{s}^{p}\right)$ is the polar component of diiodomethane SFE, $\left(\gamma_{w}\right)$ is the SFE of distilled water diiodomethane, $\left(\gamma_{w}^{p}\right)$ is the polar component of water SFE, $\left(\theta_{d}\right)$ is the contact angle (CA) of diiodomethane and $\left(\theta_{w}\right)$ is the CA of water.

The CA was measured with a goniometer on a research stand (Data Physics Instruments GmbH, Germany). The camera was used to take pictures of drops on the mortar surface. The constant volumes of water or diiodomethane drops (approx. 2 mm^3^) were applied to the surface using a micropipette. Six drops were applied onto each sample of mortar. The research was conducted at the temperature close to 22.0 °C using two liquids: distilled water and diiodomethane [59].

The profile roughness parameters were determined based on British standard BS EN ISO 4287:1998+A1:2009—Geometrical product specification (GPS). Surface texture: Profile method. Terms, definitions and surface texture parameters [60]. The roughness of the mortar surface was established by using T8000 RC120-400 (Hommel_Etamic, UK). The measurements were performed with the use of the graphical user interface (GUI), which allows to calculate the parameters of the tested roughness profiles and to assess geometrical features such as distances, maximum peaks and valleys of the tested mortar surface.

The structure of porous mortars and the hydrophobic coatings (on the mortar surface) were determined with scanning electron microscopy (SEM). The Quanta FEG 250 microscope (FEI, Hillsboro, USA) device was used for this purpose. Samples with flat surfaces were cut out of all tested mortars. The chemical composition of the mortars was analyzed using Energy Dispersive Spectroscopy (EDS) integrated with SEM. The samples for SEM investigations were glued to a carbon holder using carbon glue. Subsequently, the preparations were sprayed with a carbon layer with a wavelength of about 50 nm to obtain conductivity on the sample surface. Sample preparation methodology eliminates the possibility of micro-defections caused by the mortar surface and the hydrophobic layer cracking. In order to avoid the possibility of other surface flaws, low vacuum and low beam energy were used.

## 3. Results and Discussion

**Figure 1:**
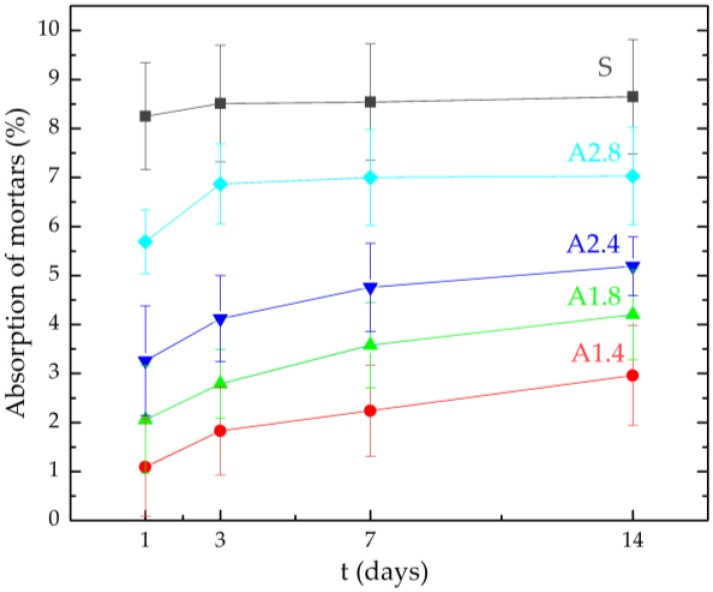
Absorption of mortars after 7, 14 and 28 days.

Table 4 and Figure 1 present mortar absorbability values after 1, 3, 7 and 14 days of testing. Surface hydrophobisation reduced mortar absorbability. The best results were obtained on samples A1.4, with higher concentrations of water-repellent preparations. After the first day of testing, water absorption decreased 7.57 times and after 14 days 2.92 times compared to reference mortars S. Hydrophobized A2.8 mortars after 14 days obtained water absorption of 7.03% and it was the largest water absorption of hydrophobized lightweight mortars. As the concentration of the hydrophobic solution increases, the absorbability decreases. Hydrophobic agent A2 at a dilution of 1:4 (sample A2.4) reduced water absorption after 14 days by 26%. However, the use of Agent A1 at the same dilution reduced the absorbability of mortars by 42%.

**Figure 2:**
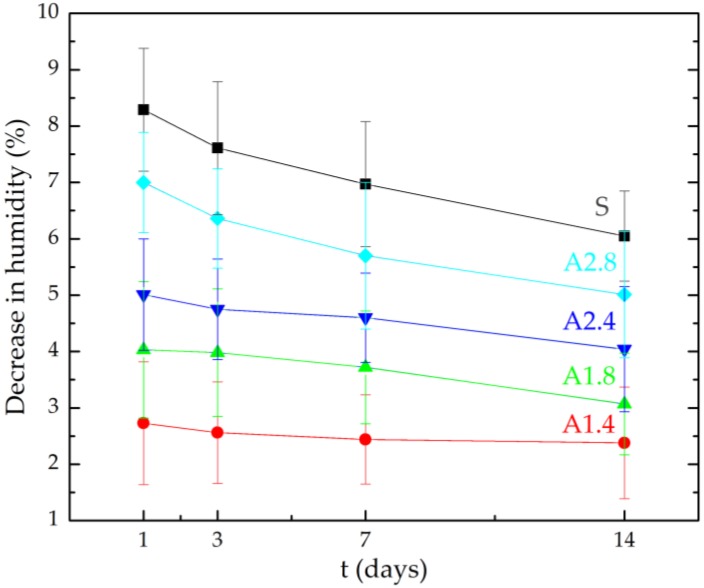
The decrease in humidity after 7, 14 and 28 days.

Table 5 and Figure 2 show the decrease in humidity after 1, 3, 7, 14 days of the study. Even though agent A1 achieved the best result when protecting the mortar from absorbability, this agent at a dilution of 1:4 slowed down the evaporation of water from the mortars the most. Only after the first day of the study, the water vapor diffusion capacity of A1.4 mortars was 67% lower and after 14 days it was 61% lower compared to non-hydrophobized mortars. Hydrophobisation prevents the evaporation of water from mortars, which has been proved in earlier works of the authors [37,44]. At a concentration of hydrophobic preparations of 1:4, the tightness of the mortar increased. As a result of which the mortars’ ability to evaporate water decreased. The effectiveness of hydrophobisation depends mainly on the porosity of the material. As the porosity increases, the absorbency of the material increases and hydrophobisation is more effective as it can reach deeper parts of the material. Błaszczyński et al. [61] noted that in the case of tight concrete, hydrophobisation proves to be less durable, as the hydrophobic preparation is not able to penetrate the structure of the composite, as it can be noticed in the case with more porous materials. Studies by Zhu et al. [62] have shown that surface hydrophobisation is more effective in reducing the capillary action than hydrophobic additives added to the mix. Moreover, it can improve resistance to chloride penetration and carbonation process.

The reference samples were tested for physical and mechanical properties. The specific and apparent density was checked. Open and total porosity was tested. Table 6 presents the results of the durability and physical properties of mortars. Numerous studies confirming the influence of expanded perlite on the physical properties of cement composites are known in the literature [63,64,65,66]

Analyzing the results, it can be unequivocally stated that the replacement of natural aggregate with expanded perlite reduces the density of mortars, which decreases as the number of perlite increases. This is due to the low specific gravity of perlite and high porosity of aggregate, which contributes to increasing the porosity of ready-mixed mortars. The mortars made by the authors were characterized by high open porosity of 14.34% and total porosity of 33.80%. Bending and compression strength tests were carried out. Due to the lack of surface hydrophobisation effect on the internal structure of mortars, density, porosity and mechanical strength were tested only on reference samples. The influence of expanded perlite on the mechanical strength of mortars has been proven for a long time. As the number of perlite increases, the bending and compressive strength in mortars decreases [67,68,69]. That is why the quantitative selection of ingredients is so important to obtain optimal values.

**Figure 3:**
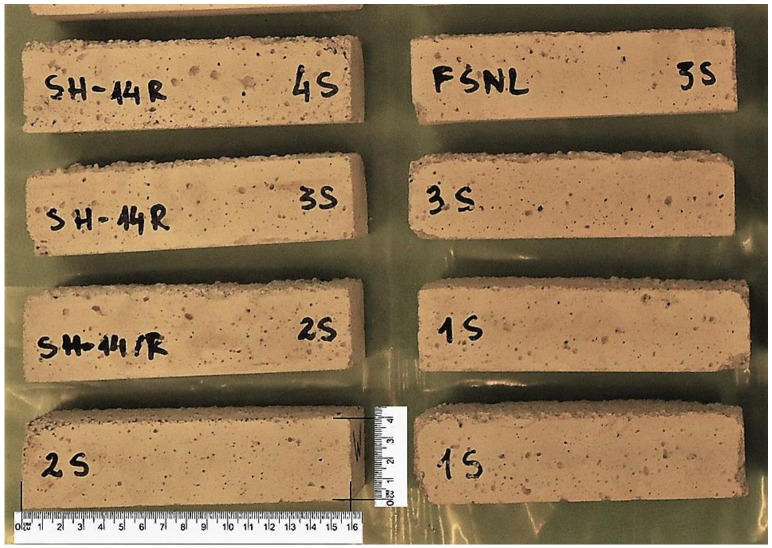
Samples after the frost resistance test.

As noted above in the case of absorbability and loss of humidity, surface hydrophobisation reduced the absorbency of mortars. The freezing water increases in volume, causing serious damage to mortars and concrete. In highly absorbent materials, more water crystallizes in their pores. The formation of an airtight coating caused difficulties in the penetration of water into the pores and thus the amount of freezing water was reduced. All the hydrophobized mortars turned out to be frost-resistant and achieved a weight loss after cyclic freezing-thaw tests of 0.06–0.19%. The lowest weight loss was observed for A1.4 mortars, where the weight loss turned out to be 34 times lower than in reference mortars S. The increase in the concentration of hydrophobic preparations caused a decrease in the weight loss of mortars. Figure 3 shows the appearance of samples after a frost resistance test.

The positive effect of surface hydrophobisation with silanes was confirmed by the studies of Barnat-Hunek et al. [70]. After 25 F-T cycles, a clear improvement in frost resistance was observed in concrete with perlite aggregate. The obtained result equal to 1.1% of weight loss was 86% lower than that of the reference mortars.

Liu Z. et al. [71] studies have proven that surface waterproofing with silane reduces damage to the concrete surface during cyclic F-T but does not prevent the occurrence of internal damage when the concrete is not sufficiently aerated. Silane forms an airtight coating on the surface of the sample making it difficult to absorb water from outside but it also hinders the diffusion of water vapor to the outside, contributing to the damage inside the sample.

Surface hydrophobisation reduced the damaging effects of salt. Hydrophobized samples proved to be resistant to salt crystallization and were characterized by a smaller mass loss of 0.10–0.43% after the test. This is due to the increased tightness of the mortars and the difficulty in absorbing salt from the solution into the internal pores. The lowest mass loss, among hydrophobized mortars, was found for samples A1.4, while the largest—A2.8. Hydrophobisation with an aqueous emulsion based on organofunctional silanes reduced weight loss by 14 and 18 times. Besides, increasing the concentration of A1 and A2 resulted in a decrease in weight loss after salt crystallization resistance test.

Sulejman et al. [72] have proven the effectiveness of the silane-based hydrophobic preparation in protecting concrete from the harmful effects of the sulphate environment. The samples were surface-coated with a hydrophobic agent and placed in 5% Na_2_SO_4_ solution. No weight change or surface damage was observed in all tested samples. The silane preparation penetrates the sample and reacts within the pores providing molecules with hydrophobic properties, making capillary action difficult and thus preventing salt crystallization in pores.

Chinese scientists have researched hydrophobic concrete with a silane-based preparation in an environment exposed to marine exposure [73]. They hydrophobized the concrete surface and placed the samples for one year in places of exposure to chloride ions from seawater. The results clearly showed that silane hydrophobic agents can inhibit the transport of chloride ions in concrete. The permeability of chloride ions after application of different silane agents decreased by 20–50% compared to reference samples without hydrophobisation.

The studies of Barnat-Hunek et.al. [37] showed that hydrophobic preparations effectively protected the surface of mortars with expanded cork, preventing its destruction. The dissolved salts had crystallized inside the samples, not damaging them, as can be seen from the increase in mass. The loss of standard mortar mass was 1.9%, while after application of alkyl-alkoxy-siloxane this loss was insignificant and amounted to 0.02%.

The contact angles (CAs) of water and diiodomethane, measured using a goniometer, are shown in Table 7.

Analyzing the results of the tests presented in Table 7, it can be noticed that the CA values depend on the type of hydrophobic agents and also on their dilution ratio (1:4 or 1:8). The highest CA of water θ_w_ = 107.5° and diiodomethane θ_d_ = 98.7° was obtained for the agents based on organofunctional silanes on dilution with water 1:4 (A1.4). For A1.8 water CA is also greater than 90°, which means that these surfaces are hydrophobic, non-wettable. In all the other cases CAs were less than 90°, which means good wetting of mortars. The effectiveness of preparation A1 was 88% and 69–81% for agent A2 depending on the dilution ratio. Mortar with A2.8 has the highest wettability after hydrophobisation, which was also shown in the study of water absorptivity (Table 4). In the literature, we can find numerous studies on the CA measurements of materials based on cement. The authors of Reference [74] examined the wettability of various porous materials using water and diiodomethane. The initial water CA of the granular materials were in the hydrophilic range because they were all less than 30°. The agent dichloro-dimethyl-silane changed the water CA from slightly greater than zero to 87.7°, indicating that it was very helpful in creating the material more hydrophobic. In another study, the CA of aggregates, that is, limestone, granite and andesite, were investigated [75]. Contact angles of 57.50°–77.79° were obtained, which indicates good absorbability of these aggregates. In contrast, Greek sandstone before hydrophobisation had a CA of 51.43° and after hydrophobisation, with organosilicon compounds (siliconates) CA was from 123° to 141°. In this case, CA was depended on the concentration of the preparation [76]. Other researchers conducted studies on the hydrophobisation of ceramic bricks from the historic Palace of the Beijing Museum [77]. Among others, silanes, siloxanes, fluorine resin was used for hydrophobising of damaged brick. Contact angles between 59° and 80° have been obtained, which is not a sign of very good hydrophobicity because the CA of the hydrophobic material should be greater than 90°. However, the non-impregnated material immediately absorbed water, which justifies the use of hydrophobisation. The other studies of mortar hydrophobisation [78] showed that the reference cement mortar was characterized by a low CA of about 37°. As a result of modifying the plasters with organosilicon compounds, which have methyl groups bonded with silicon, the phenomenon of water assuming spherical forms occurs on the mortar surface. The CA ranged from 44° to 101° and depended on the kind of hydrophobic agents. Barnat-Hunek et.al. [37] showed wide studies of surface hydrophobisation of mortar with expanded cork using the solution of methyl-silicone resin with high VOC content, low-molecular alkyl-alkoxy-silane in an organic solvent and emulsion from methyl-silicone resin in potassium hydroxide. The highest efficiency was achieved with alkyl-alkoxy-siloxanes, which caused a 2–3-fold increase in CA in light mortars. Similarly in this work, silanes (A1) caused the greatest effectiveness of hydrophobisation.

**Figure 4:**
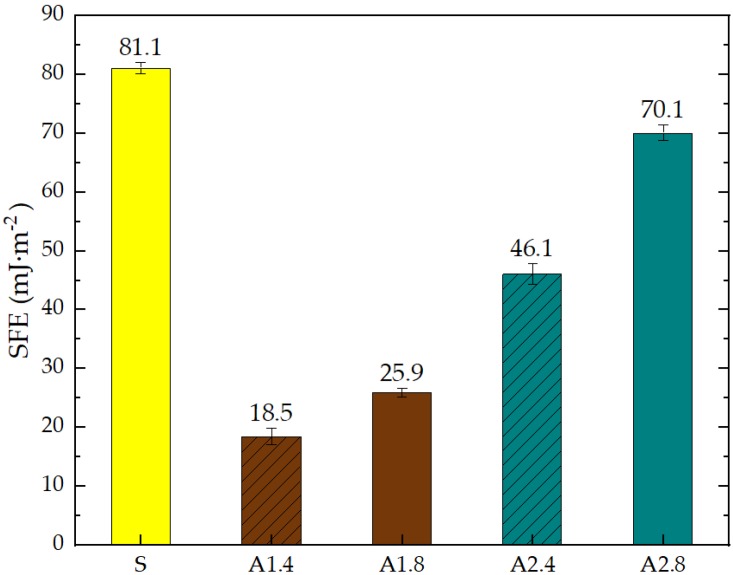
Surface free energy (SFE) of standard mortar (S) and with hydrophobising agents calculated based on Owens-Wendt method.

Based on the measured CA, SFE was determined. The SFE of standard and hydrophobic mortars calculation results are summarized in Figure 4.

Silane (A1) generated the smallest total SFE—γs = 18.5 mJ∙m^−2^ and γs = 25.9 mJ∙m^−2^ in the case of 1:4 and 1:8 dilutions, respectively. Propyl silicates (A2) has 2.5–3 times the SFE value at the same dilution as A1. Silanes have significantly reduced the adhesion of water to the mortar surface, which is associated with higher resistance to corrosive factors such as frost. The efficiency of silanes compared to standard samples was 77%. The research on the SFE of materials with cement was presented in the numerous papers [57,79,80,81]. The studies indicated that SFE for building materials surfaces ranges from 50 to 100 mJ∙m^−2^. The authors [57] demonstrated that the total SFE value, before hydrophobisation, is the highest and amounts to 76.23 mJ∙m^−2^ for high-performance concrete with granodiorite and 75.16 mJ∙m^−2^ for high-performance concrete with granite. The SFE value was to 5.5 times higher for references concrete with the granodiorite coarse aggregate and about 4 times higher for references concrete with the granite coarse aggregate than in the case of the surface hydrophobization using alkyl-alkoxy-siloxanes. Mortar tests carried out in this work confirmed the observations of other authors.

**Figure 5:**
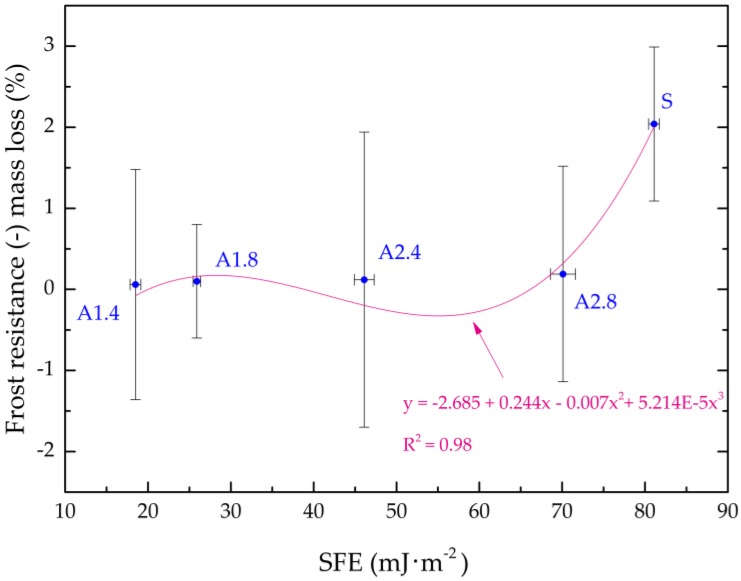
Correlation between SFE and frost resistance.

**Figure 6:**
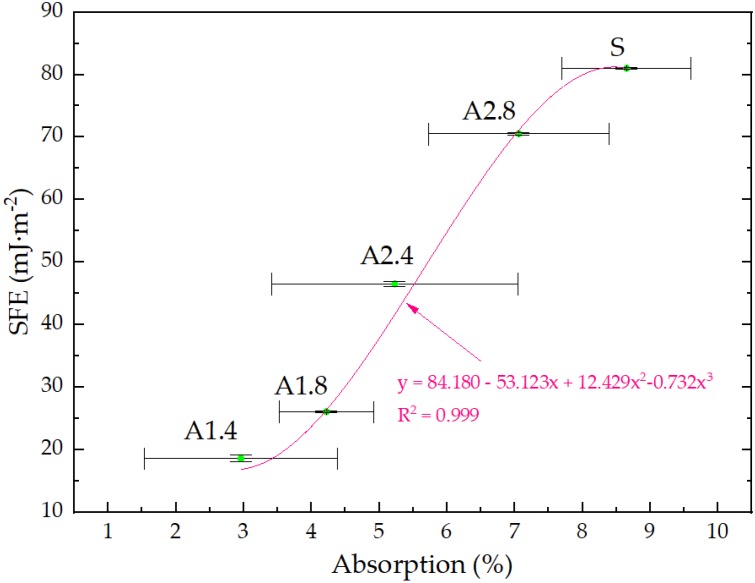
Correlation between absorption and SFE.

In our study, in order to show the relationship between the SFE and frost resistance and absorption hydrophobized and standard lightweight aggregates, a regression model (Figure 5 and Figure 6) was employed. These relations are expressed in the polynomial functions. In both cases a very good correlation coefficient (Figure 5—R^2^ = 0.98, Figure 6—R^2^ = 0.999) was obtained. These correlations primarily result from the changing porosity and structure of surface mortars depending on the hydrophobic agents used. This is because porosity is precisely connected with absorption, CA and SFE. The graphs clearly show the influence of dilution of preparations on SFE and absorption values.

The adhesion of water and salt solutions on the surface of mortars depends not only on the porosity and water absorption but also on the roughness of the material, which is proved in the next study. Mortars containing porous, lightweight aggregate, such as expanded perlite, have a slightly uneven surface that can be characterized by the following parameters:

Ra—Average Roughness; Rp—Maximum Peak Height—the maximum height of peak within evaluation length, Rv—the Maximum Valley Depth—the maximum depth observed within the evaluation length, Rmax—Maximum Peak-to-Valley Height—the maximum peak-to-valley height within any of the sampling lengths. Rmax is the sum of Rv and Rp.

The characteristics of roughness obtained for the standard and hydrophobic mortars are presented in Table 8.

These studies showed that standard mortar without hydrophobisation presented the highest characteristics of roughness. The Maximum Valley Depth (Rv), the Maximum Peak Height (Rp), as well as Average Roughness (Ra = 4.29 µm) are the highest from all mortars. This mortar is characterized also by the highest water absorption (Table 4) and highest adhesion (SFE), equal to 81.1 mJ∙m^−2^ (Figure 4). In studies of She et al. [82] a surface with low roughness, shorter peak height should exhibit a low CA between the droplet and concrete surface. Additionally, the maximum valley region contains that topographical features where water molecules absorption from the atmosphere could result in their condensation and frosting [83], what our research has shown (Table 8). The higher the mortar roughness, the greater the absorption and greater mass loss after our frost resistance test.

Meuler et al. [84] and Boinovich et al. [85] showed that the ice adhesion to a microscopically smooth surface is related to the surface’s wettability and the water-substrate adhesion (SFE). Hydrophobisation in all cases smoothed the surface of mortars. The smoothest surface was obtained by mortar A1.4. In this case, the Ra decreased 4.85 times. The concentration of A1 was high enough to seal tightly the surface pores of the samples. Higher dilution of the preparation (1:8) causes a decrease in the filling of the pores (valleys depth), the Ra increased by 15% in relation to A1.4. A2 slightly filled the surface pores.

**Figure 7:**
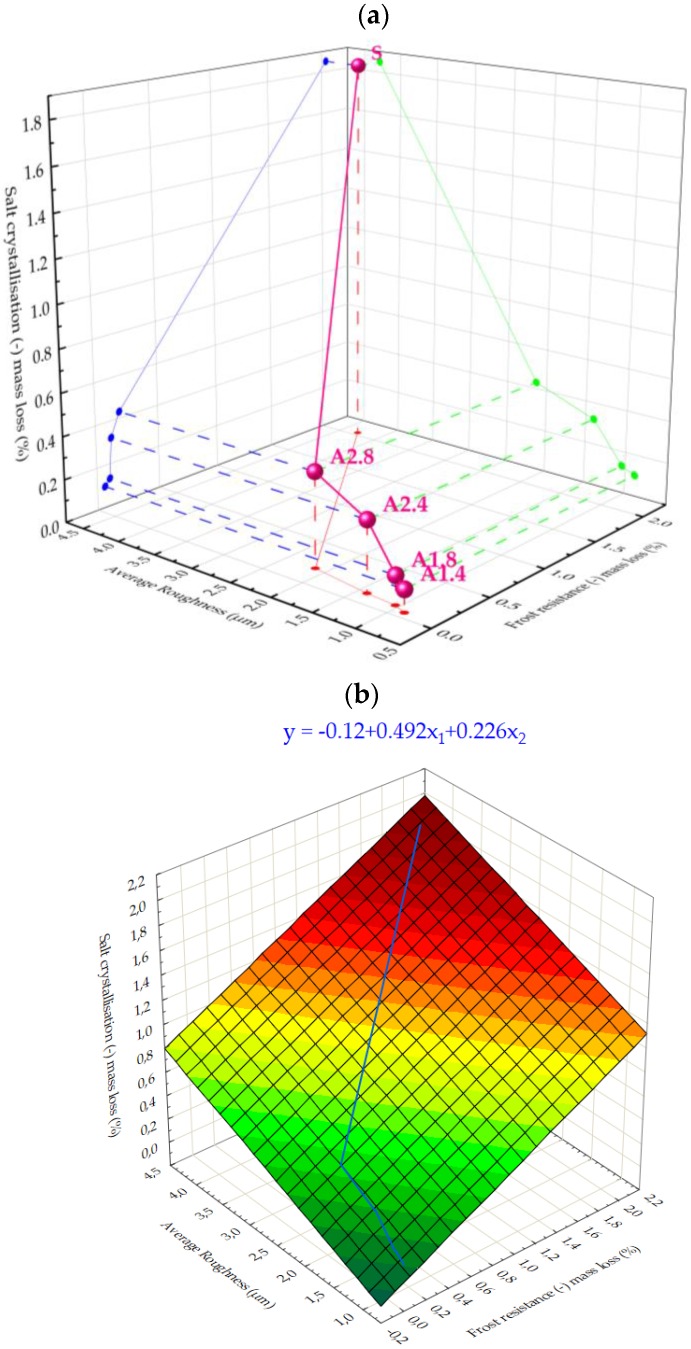
Correlation between micro-roughness, frost resistance and resistance to water-soluble salts: (**a**) 3D Scatter, (**b**) 3D Surface Plot.

Figure 7 shows a mathematical and experimental model of the mortars resistance to salt crystallization depending on two other characteristics: x_1_—frost resistance as well as x_2_—average roughness Ra. The analysis showed that the higher the roughness as well as the higher the weight loss after the frost test, the higher the weight loss after the salt test. The above model (Figure 7) presents the extent to which the characteristics of hydrophobized mortars affect their durability. Knowledge of these dependencies can be useful when selecting a suitable hydrophobic agent intended for facades exposed to frost corrosion and water-soluble salt.

**Figure 8:**
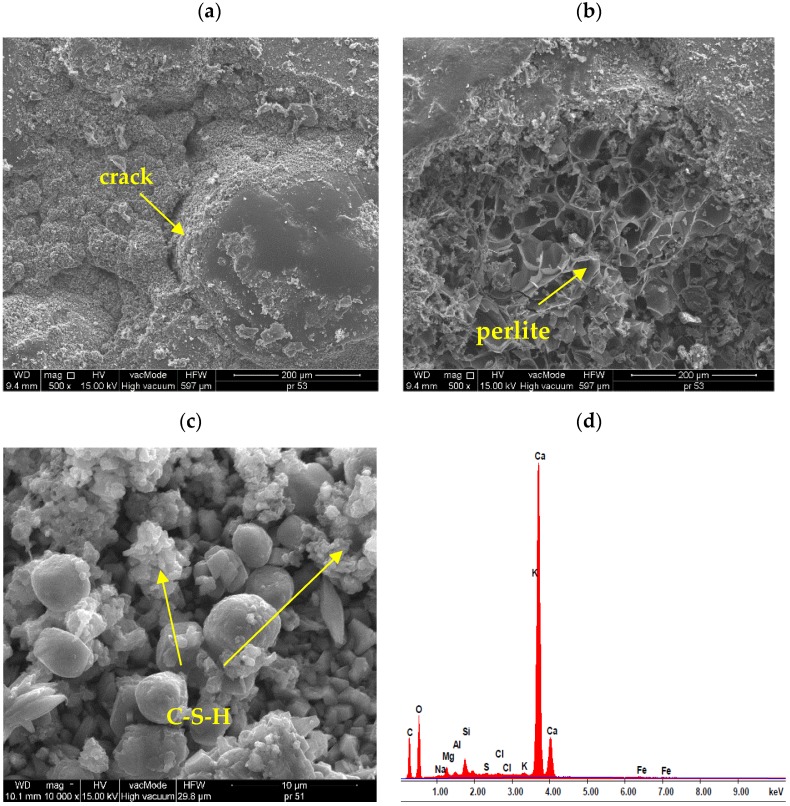
Micro-structure of standard lightweight mortars with perlite: (**a**) grain of sand surrounded by cement paste (×500), (**b**) combination of perlite with cement paste, intense interface between them (×500), (**c**) micro-area well-developed C-S-H phase crystals (×10^4^), (**d**) results of elemental analysis in Energy Dispersive Spectroscopy (EDS) micro-area from the surface of standard samples.

Figure 8 presents the SEM images of reference lightweight mortars with perlite.

The SEM studies show that in the standard mortar, hydration products mainly include spherical and irregular or flat particles that form compact aggregations. According to Diamond classification, they refer to III and IV morphological types of C-S-H phases (Figure 8c). Figure 8a shows a rather weak connection between sand grains and leaven. Cracks, gaps between the aggregate and cement paste are visible. Figure 8b shows a very good combination of paste with porous, rough aggregate - perlite. The air pores between the perlite and cement paste can influence porosity, as well as frost resistance of mortar, which was confirmed by earlier studies. Similar observations are shown on the example of mortars with expanded cork [37]. In Figure 8d, was presented results of elemental analysis in EDS micro-area from the surface of the standard sample.

The chemical composition of the analyzed lightweight mortars based on the EDS analysis was presented in Table 9. Samples for EDS analysis have been wiped from the surface itself to take into account the content of the hydrophobic coating.

The analysis of chemical composition indicates that calcium CaO, as well as silica SiO_2_ and aluminum and magnesium oxides, are found in standard and hydrophobising lightweight mortar. Out of the analyzed samples, the highest SiO_2_ content and the highest MgO content was observed in S mortar. The content of CaO is the smallest in the mortar S. None of the mortars contained P_2_O_5_. Hydrophobized mortars have very similar chemical compositions, as both preparations are silane derivatives. MgO content in mortars with the hydrophobic coating is 2.3 to 4 times lower than in S. The SO_3_ content of water-repellent mortars is 4.5 times higher than that of the reference mortar.

**Figure 9:**
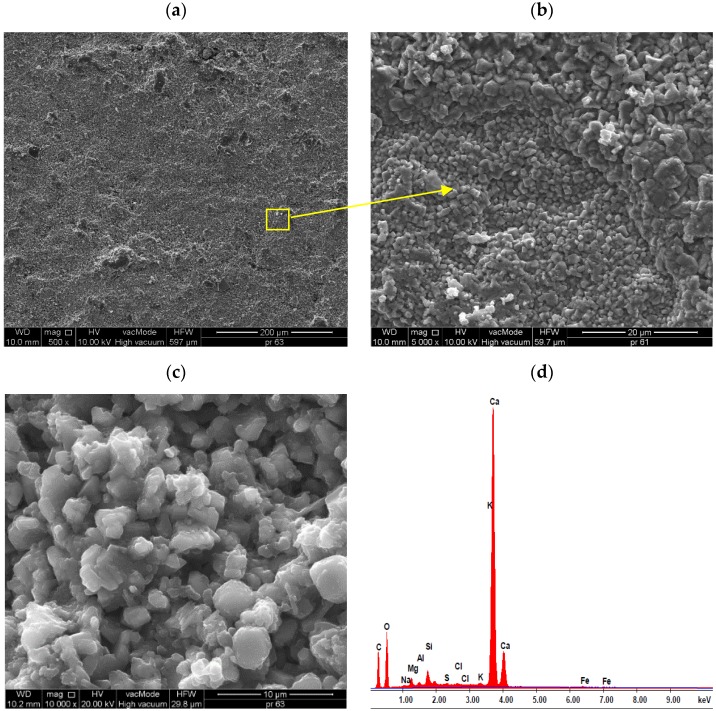
Micro-structure of hydrophobic coatings A1 on the micro-structure of mortar: (**a**) A1.4—an almost invisible thin water-repellent film that has thoroughly covered the surface of the sample (×500), (**b**) A1.4—polysiloxane gel molecules (×5000), (**c**) A1.4—micro-area well-developed C-S-H phase crystals (×10^4^), (**d**) results of elemental analysis in EDS micro-area from the surface of hydrophobized samples.

**Figure 10:**
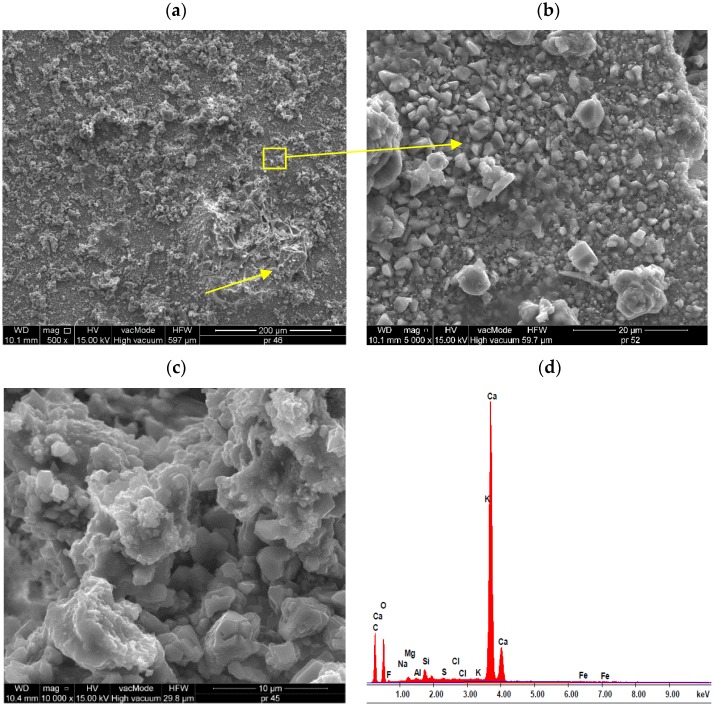
Micro-structure of hydrophobic coatings A2 on the micro-structure of mortar: (**a**) A2.8—a visible thin hydrophobic film which has not completely covered perlite grains (×500), (**b**) A2.4—polysiloxane gel particles (×5000), (**c**) A2.4—micro-area well-developed C-S-H phase crystals (×10^4^), (**d**) results of elemental analysis in EDS micro-area from the surface of hydrophobized samples.

The SEM photos (Figure 9 and Figure 10) show a very good distribution of coatings in the micro-structure of lightweight mortars.

The degree of dissolution of the hydrophobic film in the microstructure of the samples is slightly different depending on the type of preparation. In Figure 9a a thin layer is visible, which gently covered the surface of the sample without sealing it. However, Figure 10a shows that protruding sharp edges from the surface of the sample, such as perlite grain, have not been sufficiently protected with a water-repellent preparation. The open structure of the perlite contributes to greater absorbability of the samples and weaker resistance to frost and salts (Figure 7). The size of the silicone gel particle ranges from 0.5 µm to 5 μm (Figure 9b,c and Figure 10b,c). Gel particles made of A1 silane are tightly packed, adhere tightly to each other, form a compact film without cracks, which contributes to higher hydrophobic properties and lower absorption of water. In the case of preparation A2, the gel particles are dispersed, have different diameters, do not cover precisely all the components of the mortar. This results in a lower effectiveness of hydrophobisation, higher water absorption and decrease in frost resistance and resistance to salts, which was confirmed by previous studies (Table 6), as well as hydrophobized light mortars described in References [37,47]. Figure 9c and Figure 10c present micro-area well-developed C-S-H phase crystals and polysiloxane gel particles. The polysiloxane gel A1 is compact, quite tight, while coating A2 has a higher porosity. The above studies indicate that surface hydrophobisation with nano-silanes has a slight impact on cement hydration process, although cement hydration products are in all cases characteristic for III and IV morphological types of C-S-H phases. However, with the hydrophobisation of nanopolymer A1, as shown in Figure 9b,c, the surface morphology becomes denser and the pores are filled with a newly created skeleton. According to Palou et al. [86] in the earlier period of hydrothermal hardening, hydrated C-S-H products were formed during the chemical process. Due to higher crystallization pressure than in the case of modified mortars, the porosity of cement mortars increases (Figure 8c). Partial thermal decomposition of the mortars takes place under high pressure at a later stage of the curing process, which results in saturation of the sample with carbon dioxide [86]. Additionally, the silane content on the sample surface slows down the cement hydration, influencing the quality and type of produced phases, as illustrated by for example, Figure 10c. There are no visibly crystallized ettringite particles in mortars. Figure 9c and Figure 10c show a shallow rather than spherical structure of the phases, as opposed to a typical cement mortar (Figure 8c). This indicates that the silane content caused changes in the mortar structure. This is reflected in the results of physical characteristics tests, as well as in the micro-roughness, where it was shown that the coating seals the surface of the samples and changes the roughness.

## 4. Conclusions

The study investigated the effect of hydrophobisation and surface treatment of light porous mortars with perlite. This study has the following key conclusions:

- Surface hydrophobization of light mortars with perlite effectively reduced the absorbability of the samples. The best results were obtained on agent A1.4. After the first day of testing, water absorption decreased by 7.5 times and after 14 days 2.9 times compared to reference mortars S.
- Samples A1.4 were characterized by the highest tightness, in which a decrease in water vapour diffusion capacity by 61% in comparison to standard samples was observed.
- All mortars proved to be frost resistant. A decrease in weight loss was observed from 2.04% in standard samples, to 0.06–0.19% in hydrophobized mortars.
- Hydrophobized A1 and A2 mortars showed resistance to sulphate crystallisation. A decrease in weight loss after salt crystallisation from 1.85% in S samples to 0.1–0.43% in hydrophobized samples was observed.
- Samples covered with propyl silicates (A2) proved to be wettable (CA < 90°), while samples A1 showed hydrophobic properties. The highest CA equal to 107.5° was obtained in mortars A1.4. This is an almost 9-fold increase compared to standard samples S.
- Mortars A1.4 showed the smallest total SFE, equal to 18.5 mJ∙m^−2^, which is 77% lower than standard S mortars.
- The smoothest surface area was obtained in mortars A1.4, where Ra decreased 4.85 times. The study showed that the higher the roughness of the mortar, the higher the absorbency and lower the frost resistance.
- The preparation based on silane (A1) proved to be more effective in improving all properties of hydrophobized mortars. Studies have shown greater effectiveness of higher concentration preparations, diluted in water in the ratio 1:4.

Nanopolymers have proven to be effective hydrophobic agents and can be widely used to protect new and existing lightweight cement mortars exposed to aggressive environmental influences. The technologies involved in fabricating hydrophobic nanopolymers coatings should be cheaper to be more productive on a large scale. Currently, authors are during the elaboration of efficient and economical hydrophobic agents based on biopolymers, applicable at an industrial level, in order to obtain not wettable surfaces.

## Figures and Tables

**Table 1:** Composition of mortars with lightweight aggregate.

Components	Unit	S	A1.4	A1.8	A2.4	A2.8
Portland cement CEM I 42.5R	(kg∙m^−3^)	562	562	562	562	562
Sand 0–2.0 mm	(kg∙m^−3^)	1194	1194	1194	1194	1194
Perlite 0.5–2.0 mm	(kg∙m^−3^)	16.15	16.15	16.15	16.15	16.15
Water	(kg∙m^−3^)	252.9	252.9	252.9	252.9	252.9
Superplasticizer	(kg∙m^−3^)	2.2485	2.2485	2.2485	2.2485	2.2485
Dilution of hydrophobic agent	-	-	1:4	1:8	1:4	1:8

**Table 2:** Physical properties of Portland cement CEM I 42.5R [44].

Specific Surface (cm^2^∙g^−1^)	Initial Setting Time (min)	End Setting Time (min)	Specific Gravity (g∙cm^−3^)	Water Demand (%)	Compressive Strength After 2 Days (MPa)	Compressive Strength After 28 Days (MPa)
3.426	146	190	3.09	27.6	28.8	54.1

**Table 3:** Physical properties of expanded perlite.

Bulk Density (kg∙m^−3^)	Porosity (%)	Water Absorptivity (%)	Thermal Conductivity (W∙(m^2^∙K)^−1^)	Compressive Strength (N∙mm^−2^)
90	33	52	0.049	3.3

**Table 4:** Absorption of mortars (%).

Symbol of the Sample	Average Absorption (%)			
	t (days)			
	1	3	7	14
S	8.25	8.51	8.54	8.65
A1.4	1.09	1.83	2.24	2.96
A1.8	2.06	2.79	3.58	4.20
A2.4	3.26	4.12	4.76	5.19
A2.8	5.69	6.87	7.00	7.03

**Table 5:** The decrease in humidity (%).

Symbol of the Sample	Average Humidity (%)			
	t (days)			
	1	3	7	14
S	8.29	7.61	6.97	6.05
A1.4	2.73	2.56	2.44	2.38
A1.8	4.03	3.98	3.72	3.07
A2.4	5.01	4.75	4.60	4.04
A2.8	7.00	6.36	5.70	5.01

**Table 6:** Durability and physical properties of lightweight mortars.

Durability/ Physical Properties	Unit	S	A1.4	A1.8	A2.4	A2.8
Apparent density	(g∙mm^−3^)	1.70	-	-	-	-
Specific density	(g∙mm^−3^)	2.55	-	-	-	-
Porosity	(%)	14.34	-	-	-	-
Total porosity	(%)	33.80	-	-	-	-
Flexural strength	(N∙mm^−2^)	5.6	-	-	-	-
Compressive strength	(N∙mm^−2^)	37.3	-	-	-	-
Frost resistance (-) mass loss	(%)	2.04	0.06	0.10	0.12	0.19
Salt crystallisation (-) mass loss	(%)	1.85	0.10	0.13	0.32	0.43

**Table 7:** Angles of liquids of standard and hydrophobized mortars.

Type of Mortar	Contact Angle	
	Water *θ_w_* (°)	Diiodomethane *θ_d_* (°)
S	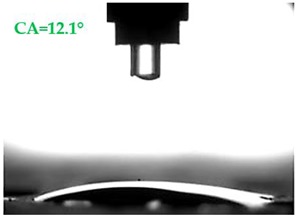	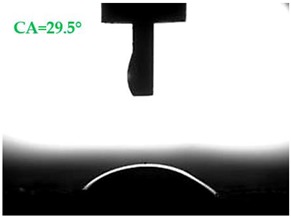
A1.4	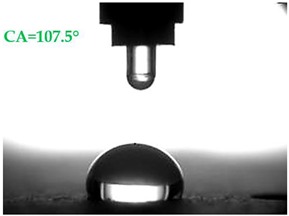	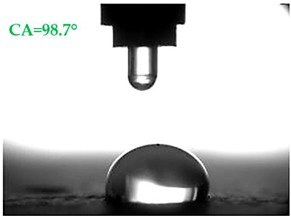
A1.8	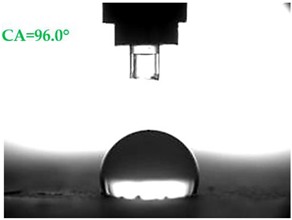	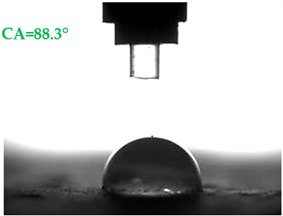
A2.4	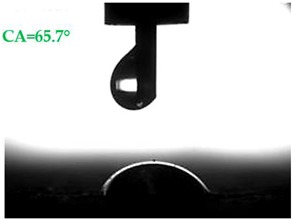	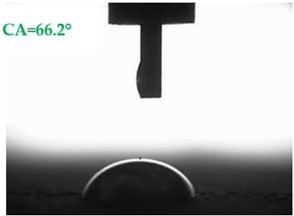
A2.8	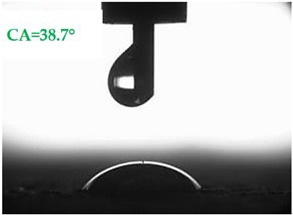	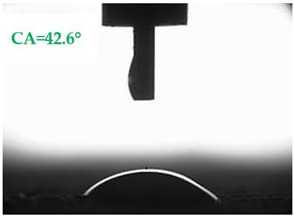

**Table 8:** Micro-roughness characteristics and image of the topography of mortars.

	Micro-Roughness Characteristics (µm)	
S	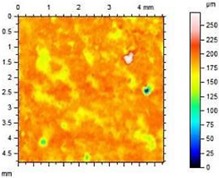	R_a_ = 4.29 µm R_p_ = 10.3 µm R_v_ = 10.6 µm R_max_ = 20.9 µm
A1.4	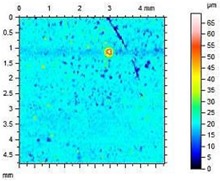	R_a_ = 0.883 µm R_p_ = 4.21 µm R_v_ = 3.01 µm R_max_ = 20.9 µm
A1.8	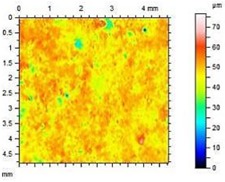	R_a_ = 1.04 µm R_p_ = 3.09 µm R_v_ = 4.24 µm R_max_ = 7.33 µm
A2.4	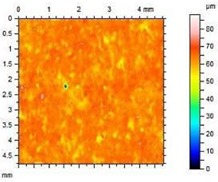	R_a_ = 1.40 µm R_p_ = 4.57 µm R_v_ = 4.75 µm R_max_ = 9.32 µm
A2.8	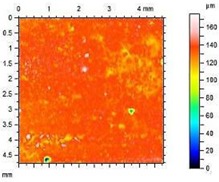	R_a_ = 2.13 µm R_p_ = 6.84 µm R_v_ = 6.33 µm R_max_ = 13.17 µm

**Table 9:** The chemical composition of the standard and hydrophobized lightweight.

Mortar		Compound								
		Al_2_O_5_	SiO_2_	Na_2_O	Fe_2_O_3_	MgO	K_2_O	CaO	SO_3_	P_2_O_5_
S	Content (%mass)	2.30	9.77	1.17	0.50	10.0	0.56	74.93	0.38	-
A1.4		3.06	6.91	1.51	0.65	4.22	0.86	80.15	1.74	-
A2.4		2.57	5.60	1.25	0.46	2.50	0.71	80.96	1.71	-
